# Phase II study of bevacizumab and erlotinib in the treatment of advanced hepatocellular carcinoma patients with sorafenib-refractory disease

**DOI:** 10.1007/s10637-012-9808-8

**Published:** 2012-03-09

**Authors:** Thomas Yau, Hilda Wong, Pierre Chan, T. J. Yao, R. Pang, T. T. Cheung, S. T. Fan, Ronnie T. Poon

**Affiliations:** 1 Department of Surgery, The University of Hong Kong, Queen Mary Hospital, Room 211B, 2/F New Clinical Building, 102 Pokfulam Road, Hong Kong, China; 2 Department of Medicine, The University of Hong Kong, Queen Mary Hospital, Hong Kong, China; 3 Clinical Trials Centre, The University of Hong Kong, Queen Mary Hospital, Hong Kong, China; 4 State Key Laboratory of Liver Diseases, The University of Hong Kong, Queen Mary Hospital, Hong Kong, China

**Keywords:** Bevacizumab, Erlotinib, Advanced HCC, Sorafenib-refractory

## Abstract

*Background* The combination of bevacizumab (B) and erlotinib (E) has shown promising clinical outcomes as the first-line treatment of advanced HCC patients. We aimed to evaluate the efficacy and safety of using combination of B + E in treating advanced HCC patients who had failed prior sorafenib treatment. *Methods* Eligible advanced HCC patients with documented radiological evidence of disease progression with sorafenib treatment were recruited. All patients received bevacizumab(B) at 10 mg/kg every 2 weeks with erlotinib(E) at 150 mg daily for a maximum of 6 cycles. Response assessments using both RECIST and modified RECIST criteria were performed after every 6 weeks. The primary endpoint was clinical benefit (CB) rate and a Simon two-stage design was employed. *Results* The trial was halted in the first stage according to the pre-set statistical criteria with 10 patients recruited. The median age was 47 years (range, 28–61) and all patients were in ECOG performance status 1. Eighty percent of patients were chronic hepatitis B carriers and all patients had Child A cirrhosis. Among these 10 patients, none of the enrolled patients achieved response or stable disease. The median time-to-progression was 1.81 months (95 % confidence interval [C.I.], 1.08–1.74 months) and overall survival was 4.37 months (95 % C.I., 1.08–11.66 months). Rash (70 %), diarrhea (50 %) and malaise (40 %) were the most commonly encountered toxicities. *Conclusion* The combination of B + E was well tolerated but had no activity in an unselected sorafenib-refractory advanced HCC population. *Condensed abstract* The combination of bevacizumab and erlotinib had no clinical activity in sorafenib-refractory HCC population.

## Introduction

Hepatocellular carcinoma (HCC) is an aggressive cancer with poor prognosis, representing the third commonest cause of cancer mortality worldwide [1]. The majority of HCC patients present with advanced disease not amenable to loco-regional therapy [2]. In these patients, systemic treatment with chemotherapy, immunotherapy or hormonal therapy result in low response rates and no survival benefit [3].

More recently, increased understanding of the molecular biology of HCC has facilitated rational development of therapeutic targeted agents. Tumor angiogenesis plays a pivotal role in the development and progression of HCC; increased expression of vascular endothelial growth factor (VEGF) is frequently observed in these tumors [4, 5]. Sorafenib, an oral multi-kinase inhibitor, exerts anti-angiogenic and anti-tumor effects by blocking multiple growth factor pathways including VEGF receptor (VEGFR)-1, -2, -3, platelet derived growth factor receptor (PDGFR)-B, RAF, RET and FLT-3[6]. Two pivotal phase III randomized trials conducted respectively in the Western [7] and Asian [8] populations have demonstrated significant survival improvement with single agent sorafenib in treating advanced HCC patients, leading to the approval for use of sorafenib in these patients. On the other hand, bevacizumab (B), a recombinant humanized monoclonal antibody that binds VEGF [9], is still under investigation in HCC. Response rates of 13 % as monotherapy [10] and of 11–20 % when combined with chemotherapy [11–13] were reported in phase II trials.

In addition to angiogenesis, the epidermal growth factor (EGF) and its receptor (EGFR) also play a crucial role in the proliferation of HCC [14, 15]. Single agent erlotinib (E), an EGFR tyrosine kinase inhibitor, achieved modest clinical benefit in the management of advanced HCC patients in the phase II setting [16, 17].

These data provide the rationale for evaluating the combination of B + E in advanced HCC. Two phase II studies have demonstrated benefit of the combination in patients who had not received prior anti-VEGF or anti-EGFR agents [18, 19]. The promising first-line activity of B + E confirms the importance of VEGF and EGFR pathways in HCC. On the other hand, there is currently no standard systemic therapy for patients who progress after sorafenib. Therefore, in this study, we aimed to evaluate the efficacy and safety of the combination of B + E in treating advanced HCC patients who had failed first-line sorafenib treatment.

## Patients and methods

This was an open-label, prospective, single arm pilot study to investigate the efficacy and safety of B + E combination in advanced HCC patients who progressed after prior sorafenib treatment. The protocol was approved by the institutional ethic committee and written consents were obtained from the patients before enrollment.

### Patient’s eligibility

Advanced HCC patients, who were not suitable for surgery or various loco-regional therapies at the Queen Mary Hospital, Hong Kong were enrolled. HCC was diagnosed either by cyto-histological confirmation or by non-invasive criteria according to the European Association for Study of Liver disease (EASL) criteria. Staging was by both America Joint Committee on Cancer (AJCC) and Barcelona Clinic Liver Cancer (BCLC) staging. All the enrolled patients had documented radiological evidence of disease progression with sorafenib treatment. Moreover, all patients had washout period of about 2 weeks but not longer than 4 weeks after the last sorafenib dosing. Other major eligibility criteria included adult patients aged ≥18 years; patients with Child-Pugh class A cirrhosis; Eastern Cooperative Oncology Group (ECOG) performance status 0–1; expected life expectancy of ≥12 weeks and with adequate organ function. Moreover, the disease must be measurable with at least 1 lesion, which is at least 1 cm in 1 dimension on computer tomography (CT) or magnetic resonance imaging (MRI) scan. Major exclusion criteria included prior anti-VEGF therapy other than sorafenib.

### Treatment design and disease evaluation

All patients received B at 10 mg/kg every 2 weeks together with E at 150 mg daily for a maximum of 6 cycles.

A full history and clinical examination were performed at every clinical visit. Disease assessment was performed by CT scan every 3 cycles i.e. 6 weeks. MRI and positron emission tomography (PET) with choline-acetate as radio-isotope were performed only in case of diagnostic uncertainty. Response was determined by independent radiologists and classified according to both RECIST 1.0 [20] and modified RECIST criteria [21]. All patients who had received at least one cycle of treatment were considered evaluable for tumour response and safety. Toxicities were evaluated according to National Cancer Institute (NCI)’s Common Terminology Criteria for Adverse Events (CTCAE) version 3.0. Patients who had either complete response (CR), partial response (PR) or stable disease (SD) were classified as having clinical benefit (CB) and continued the regimen for another 3 cycles. After six cycles, only patients had CB continued with erlotinib until progressive disease (PD) or intolerable toxicities.

### Safety monitoring

Safety assessments consisted of monitoring and recording all the adverse events and serious adverse events throughout the study period. Apart from monitoring of vital signs, regular collection of urine, hematology and blood chemistry of the enrolled subjects were performed. All patients who had received at least one cycle of treatment were considered evaluable for safety.

### Endpoints

The primary endpoint was CB rate. The secondary endpoints included response rate (RR), serial serum alpha fetoprotein (AFP) measurements, time to progression (TTP), overall survival (OS) and safety.

### Statistical analysis

The null hypothesis was that B + E would give a CB rate of no more than 5 %, and the alternative hypothesis was that the CB was no less than 20 %. Based on Simon’s optimal two stage design with 5 % maximal tolerable false positive rate and 20 % maximal tolerable false negative rate, 10 patients would be enrolled at stage one. If no patient shows any CB, the study would be terminated, with the conclusion that the CB rate was ≤5 %. If one or more patients derived CB, then up to 29 patients in total would be enrolled. If more than three patients had CB, we then rejected the null hypothesis and considered B + E sufficiently promising to warrant further study.

Survival analysis was computed by the Kaplan-Meier method. TTP was calculated from the date of commencement of study drugs to the date of documented progression or death. OS was calculated from the date of commencement of study drugs to the date of death or last follow-up. The analysis was performed on intent-to-treat basis. All statistical analyses were performed by R version 2.13.2 for Windows.

## Results

### Patient demographics

Between August, 2007 and May, 2008, 10 patients were recruited at first stage. They all received B at 10 mg/kg every 2 weeks together with E at 150 mg for the treatment of advanced HCC after sorafenib failure. Table 1 shows the demographic data of these patients. The median age was 47 years (range, 28–61 years) and the majority was male patients (70 %). All the enrolled patients had ECOG performance status 1. Eight (80 %) patients were chronic hepatitis B carriers and none of them were chronic hepatitis C carriers. Notably, all recruited patients had underlying Child cirrhosis. All patients had advanced disease at the time of enrollment with half of the patients had elevated alpha-fetal protein (AFP) level. Nearly all patients were in BCLC stage C disease except one. The commonest site of metastases was lung (60 %) and main portal vein invasion was present in three patients (30 %). Four patients had received prior liver resection for HCC but none of the patients had undergone liver transplantation. Three enrolled patients had received prior palliative radiotherapy for the treatment of advanced HCC. All the enrolled patients had received single agent sorafenib as the first-line systemic treatment for advanced HCC. The median duration of prior sorafenib was 2.76 months (range, 2.12–6.18 months). None of these ten patients had demonstrated major treatment response to prior sorafenib treatment. They were all confirmed to have developed radiological progression with sorafenib treatment.

**Table 1:** Demographic data of the enrolled patients in the study

Characteristics	
Age (years)	
Median	47
Range	28–61
Sex	
Male	7 (70 %)
Female	3 (30 %)
ECOG	
0	0 (0 %)
1	10 (100 %)
Hepatitis Serology	
Hep Bs Ag positive	8 (80 %)
Anti-HCV Ab positive (*n* = 8)	0 (0 %)
Child-Pugh Status	
A	10 (100 %)
B	0 (0 %)
C	0 (0 %)
Alpha-fetal Protein (AFP)	
≤400	5 (50 %)
>400	5 (50 %)
Disease Stage at the Time of Study Entry	
AJCC Staging	
I	0 (0 %)
II	0 (0 %)
IIIA	3 (30 %)
IIIB	0 (0 %)
IIIC	0 (0 %)
IV	7 (70 %)
BCLC	
A	0 (0 %)
B	1 (10 %)
C	9 (90 %)
D	0 (0 %)
Distant Metastases	
Lung	6 (60 %)
Bone	2 (20 %)
Adrenal	0 (0 %)
Spleen	1 (10 %)
Brain	1 (10 %)
Invasion of Major Vessels	
Main portal vein invasion	3 (30 %)
Hepatic vein invasion	0 (0 %)
Inferior vena cava invasion	2 (20 %)
Prior Treatment	
Surgical Treatment	
Liver resection	4 (40 %)
Liver transplantation	0 (0 %)
Local Ablative Procedures	
TACE	2 (20 %)
RFA	1 (10 %)
Systemic Therapy	
Sorafenib	10 (100 %)
Doxorubicin	1(10 %)
Radiotherapy	3 (30 %)

*Hep Bs Ag* Hepatitis B surface antigen; *Anti-HCV Ab* Anti-hepatitis C antibody; *TACE* Transarterial chemo-embolization; *RFA* Radiofrequency ablation; *AJCC* America Joint Committee on Cancer; *BCLC* Barcelona Clinic Liver Cancer; *TACE* Transarterial chemoembolisation; *RFA* Radiofrequency ablation

### Treatment efficacy and survival analysis

Table 2 summarizes the efficacy and the survival analysis of the enrolled patients. Two patients only received 2 cycles of B + E and died rapidly due to disease progression without formal radiological assessment for treatment response.

**Table 2:** Treatment efficacy and survival analysis

Duration of Prior Sorafenib Treatment (months)	
Median (range)	2.76 (2.12–6.18)
Number of B + E Treatment Cycles	
1	0 (0 %)
2	2 (20 %)
3	8 (80 %)
Overall Survival (months)	
Median (95 % C.I.)	4.37 (1.08, 11.66)
Progression-free Survival (months)	
Median (95 % C.I.)	1.51 (1.08, 1.74)

**Fig. 1:**
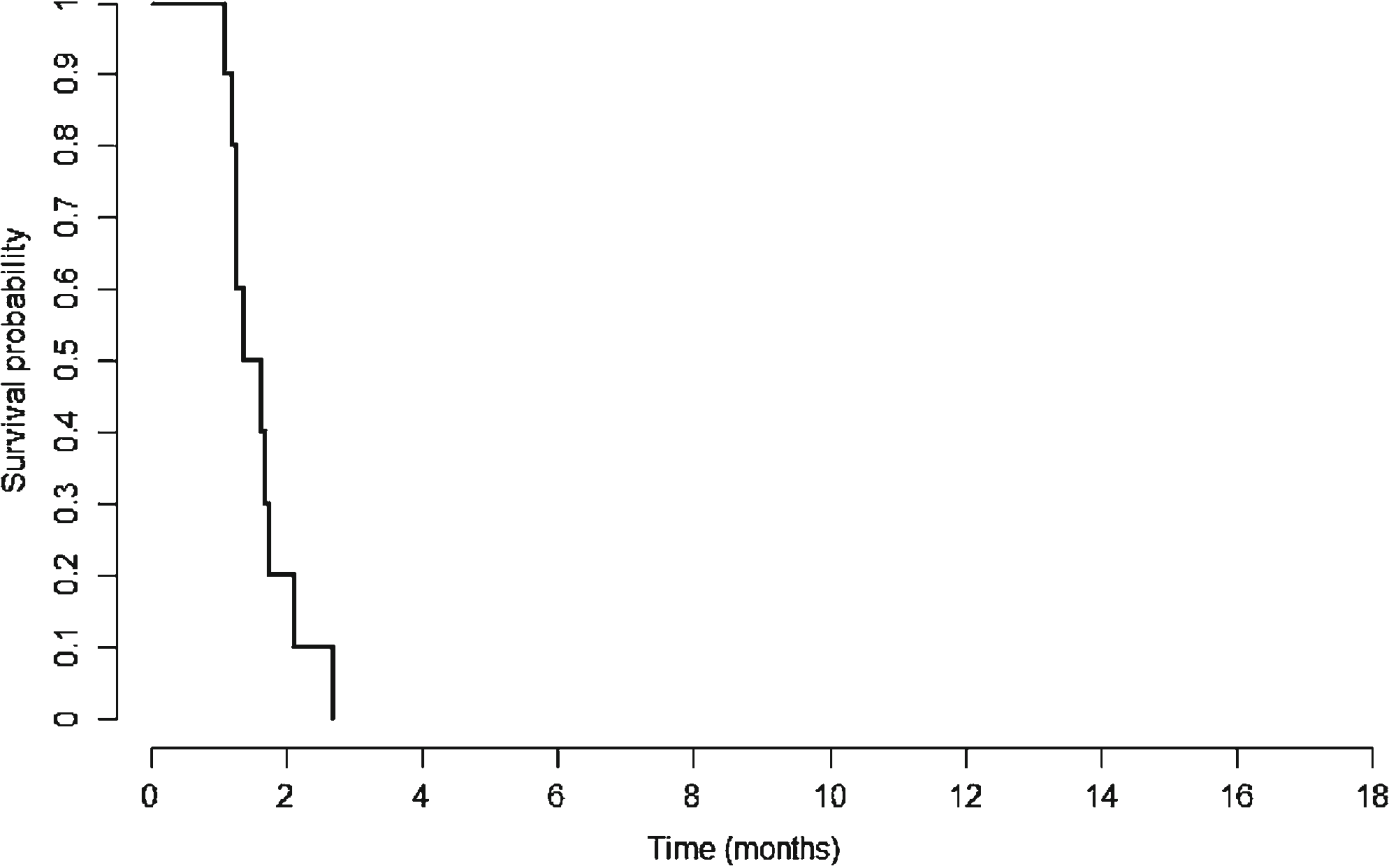
Kaplan-Meier curve of time to progression of the enrolled patients

**Fig. 2:**
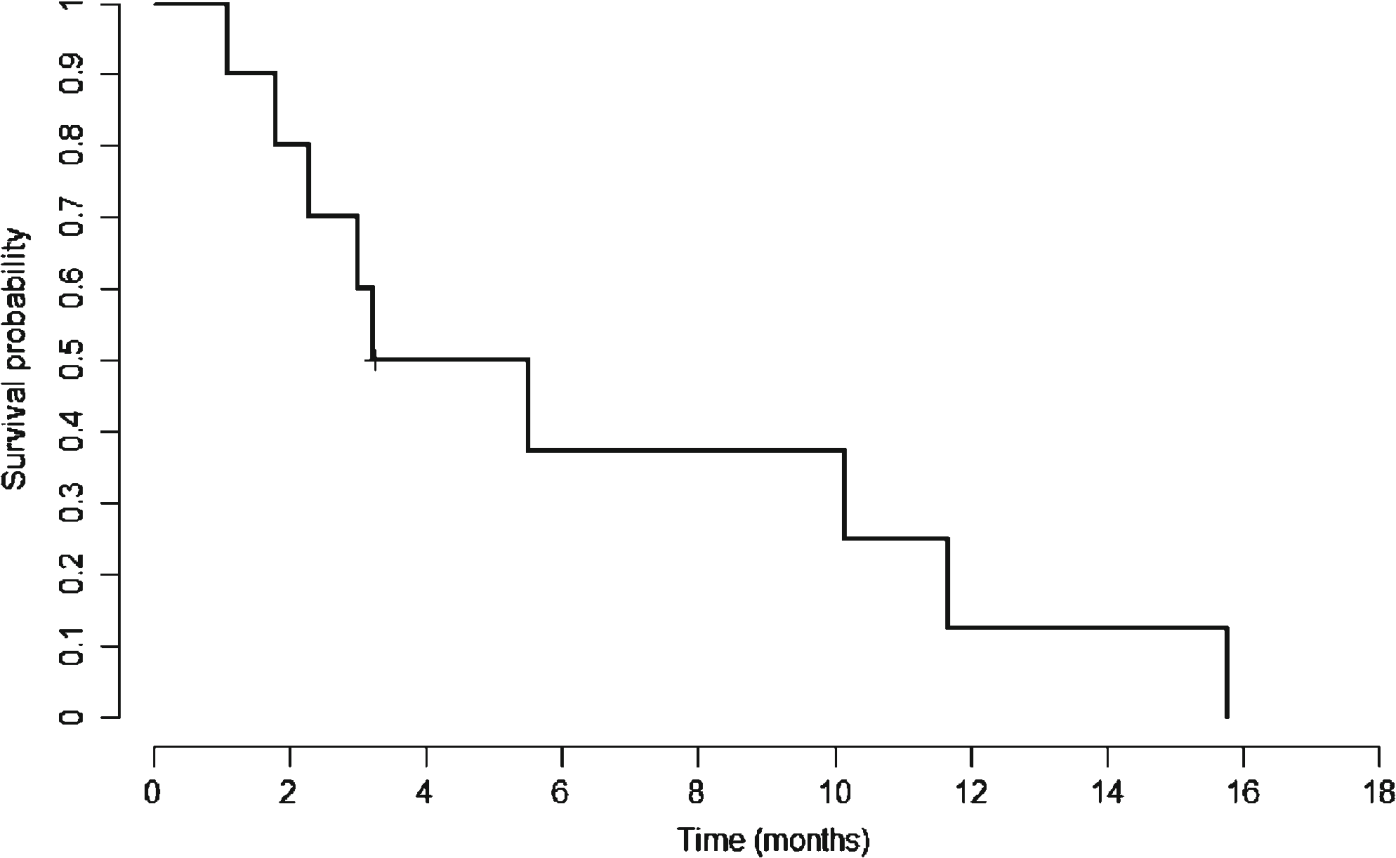
Kaplan-Meier curve of overall survival of the enrolled patients

The overall response rate was 0 % with no CR or PR observed in the enrolled cohort according to either RECIST 1.0 or modified RECIST criteria. Moreover, none of the enrolled patients had demonstrated SD. There was no significant drop in AFP level (.20 %) compared to the baseline in all the enrolled patients. The overall CB rate was 0 %. The median TTP was 1.81 months (95 % confidence interval [C.I.], 1.08–1.74 months) and OS was 4.37 months (95 % C.I., 1.08–11.66 months) (Figs. 1, 2). Thus, the primary endpoint of the study was not met and it was halted in the first stage according to the pre-set statistical criteria, with a conclusion that the CB rate to the B + E combination for sorafenib refractory patients was no more than 5 %.

### Treatment-related toxicities

Table 3 shows the details of treatment-related toxicities in the enrolled patients. Grade 3 or 4 non-hematological toxicities were reported in 20 % of the enrolled patients. Regarding the bleeding risk, only one enrolled patient experienced grade 3 bleeding from the upper gastrointestinal tract. Overall, two patients had transient treatment interruption due to grade 3 non-haematological toxicities and none of the enrolled patients died of treatment-related complications.

**Table 3:** Summary of toxicities associated with Bevacizumab and Erlotinib combination

Toxicity	Any grade	Grade 1–2 (%)	Grade 3 (%)	Grade 4 (%)
Non-haematological				
Diarrhea	5 (50 %)	4 (40 %)	1 (10 %)	0 (0 %)
Malaise	4 (40 %)	4 (40 %)	0 (0 %)	0 (0 %)
HFSR	0 (0 %)	0 (0 %)	0 (0 %)	0 (0 %)
Alopecia	2 (20 %)	2 (20 %)	0 (0 %)	0 (0 %)
Rash	7 (70 %)	6 (60 %)	1 (10 %)	0 (0 %)
Abdominal pain	2 (20 %)	2 (20 %)	0 (0 %)	0 (0 %)
Hypertension	1 (10 %)	1 (10 %)	0 (0 %)	0 (0 %)
Nausea	2 (20 %)	2 (20 %)	0 (0 %)	0 (0 %)
Mucositis	1 (10 %)	0 (0 %)	1 (10 %)	0 (0 %)
Constipation	1 (10 %)	1 (10 %)	0 (0 %)	0 (0 %)
Vomiting	1 (10 %)	0 (0 %)	1 (10 %)	0 (0 %)
Haematological				
Thrombocytopenia	2 (20 %)	2 (20 %)	0 (0 %)	0 (0 %)
Neutropenia	0 (0 %)	0 (0 %)	0 (0 %)	0 (0 %)
Leukocytopenia	0 (0 %)	0 (0 %)	0 (0 %)	0 (0 %)
Anemia	1 (10 %)	1 (10 %)	0 (0 %)	0 (0 %)
Biochemical				
Hyperbilirubinemia	0 (0 %)	0 (0 %)	0 (0 %)	0 (0 %)
ALT	2 (20 %)	1 (10 %)	1 (10 %)	0 (0 %)
AST	2 (20 %)	1 (10 %)	1 (10 %)	0 (0 %)

## Discussion

In the past few decades, there has been a step forward in the treatment of advanced HCC. Sorafenib has demonstrated modest activity in improving the clinical outcome of this lethal disease. To date, it is the only approved targeted agent for this indication. On the other hand, the absolute magnitude of clinical benefit in terms of OS associated with sorafenib was only 2–3 months [7, 8]. Furthermore, all patients on sorafenib eventually progress on the treatment. The growing sorafenib-refractory patient population is therefore an important clinical problem and the development of second-line treatment is urgently needed in the HCC community. The current study explores the role of further biological pathway inhibition after sorafenib failure; it is the first reported in the literature evaluating the combination of B and E in patients who had received prior sorafenib treatment. The results of the present study showed that B + E was well tolerated in advanced HCC patients previously treated by sorafenib and/or other therapies. However, it did not demonstrate any signal of activity in using B + E combination in treating advanced HCC progressed after sorafenib failure.

Thus far in the literature, there are only two published studies in evaluating the efficacy of B + E in treating advanced HCC patients and all these studies were tested in the patients naïve to anti-VEGF therapies. An impressive RR of 25 %, median progression free survival (PFS) of 9 months and OS of 15.65 months were reported in a single institute phase II trial which included 40 advanced HCC patients [18]. This trial had excluded patients who had prior anti-VEGF or anti-EGFR agents, although 20 % of the enrolled patients had been treated with one line of systemic chemotherapy. Notably, with longer follow-up period and recruitment of more patients, the overall RR (28 %) and CB rate (90 %) were maintained. Nevertheless, the PFS (7.9 months) and OS (12.8 months) were not as impressive as the results shown in the initial report [22]. On the other hand, Philip et al. recently reported the results of a multi-institutional phase 2 study with similar design to the aforesaid study; only one out of 27 patients had PR while 11 % had SD, with median PFS of 3 months and OS of 9.5 months [19]. In distinct contrast to the study performed by Thomas et al. [18], the results of the phase 2 study performed by Philip et al. did not support the activity in using B + E combination in treating advanced HCC patients who were naïve to anti-VEGF therapy. In fact, the combination of B + E with different dosing schedules of B has also been tested in various solid malignancies with poor results. Ko et al. had performed the phase II study in testing the use of B + E in the treatment of gemcitabine-refractory advanced pancreatic cancer with disappointing results [23]. Dickler et al. also showed that the combination of B + E had very limited activity in treating unselected patients with metastatic breast cancer [24]. Similarly, the combination has minimal clinical activity in patients with advanced upper gastrointestinal cancers [25] and recurrent advanced squamous cell carcinoma of the head and neck [26]. All the aforementioned studies consistently demonstrated that although B + E was a fairly well tolerated regime, it had minimal clinical activity in various solid malignancies.

The mechanisms of resistance to sorafenib in HCC are still poorly understood. Proposed mechanisms of sorafenib resistance include enhanced alternative pro-angiogenic signaling, which may result either from an upregulation of alternative pathways or from the pre-existence of multiple redundant signals [27]. Importantly, increased capabilities for proliferation and invasion without angiogenesis may also be observed in resistance to anti-VEGF therapies [27]. Thus, continued anti-angiogenic therapy with B, combined with E inhibiting an additional essential tumor growth pathway in HCC, and may potentially circumvent sorafenib resistance. Nonetheless, our results showed that the combination has no activity after sorafenib failure. The negative results may be explained by few reasons. First, tumour dependence on pro-angiogenic factors may be altered after sorafenib treatment [28]. The vascular remodeling due to pericytic over-coverage renders the neovasculature less responsive to VEGF for growth dependence effectively circumventing a blocked signaling pathway with greater dependence on other alternate mechanisms [29]. Second, it has been shown that the inhibition of VEGF receptors may result in an increased propensity for metastatic dissemination as the hypoxic microenvironment associated with the sorafenib use selects for highly aggressive, invasive tumor cells [30]. Therefore, the increase in the biological aggressiveness of the tumor following sorafenib resistance may account for the poor response to subsequent systemic therapy. Third, other factors including fibroblast growth factors (FGFs), insulin like growth factors (IGFs), angiopoietins, and tumor-stromal interaction may contribute to sorafenib resistance [27]. These may enable tumors to evade inhibition by B + E. Last but not least, vascular remodeling may play a significant role in the resistance to B + E. Tumors that grow during sorafenib treatment developed as viable clusters surrounding strikingly remodeled vessels. These vessels display significant increases in active proliferation of vascular mural cells, expression of platelet-derived growth factor-B and ephrinB2. Thus, enhanced vascular stability accounts for the poor response to B + E combination [31].

There are important limitations in the current study needed to be addressed. Firstly, none within our patient cohort had derived major tumor response while on sorafenib, suggesting that these patients might be intrinsically resistant to any anti-angiogenic strategies. Whether B + E is active in patients with initial response but acquired resistance to sorafenib cannot be concluded from the current study. Secondly, the relative small number of patients recruited from a single institute in the present study is noteworthy. Thus, our present findings should be regarded as exploratory rather than confirmatory. Last but not least, the safety profile of the combination reported from this study is based on a very limited number of enrolled patients, thus these data should be regarded as preliminary and interpreted cautiously. There are few ongoing trials both in US and Asia for investigating the potential benefits in employing B + E in treating advanced HCC patients. In particular, a single arm phase II trial is currently conducted in MD Anderson Cancer Centre in evaluating the role of B + E as a second-line therapy in patients who have progressed after first-line sorafenib treatment [32]. The final results of this study will be eagerly awaited to provide more data about the benefits of B + E in advanced HCC patients progressed after sorafenib treatment. More importantly, the development of biomarkers to select patients likely to benefit from targeted therapy cannot be over-emphasized. In a recent study, high VEGF expression was correlated to longer OS and high VEGFR-2 expression to shorter PFS in upper GI cancers treated with B + E, while EGFR expression and KRAS mutation status were not predictive [33]. Prospectively designed clinical trials with rigorous tissue sampling are desperately needed.

In conclusion, the combination of B + E is inactive in an unselected population of sorafenib-refractory advanced HCC patients. Further evaluation of this combination in biomarker-selected patients may be warranted.
